# Successful debulking of plurihormonal pituitary macroadenoma with long‐acting pasireotide and dopamine agonist combination therapy

**DOI:** 10.1002/ccr3.1961

**Published:** 2019-01-28

**Authors:** Terri W. Jerkins, Randy K. Jerkins, Robbi Franklin

**Affiliations:** ^1^ Midstate Endocrinology Associates Nashville Tennessee; ^2^ College of Pharmacy Lipscomb University Nashville Tennessee; ^3^ Nashville Neurosurgery Group Nashville Tennessee

**Keywords:** endocrinology, metabolic disorders, pasireotide and bromocriptine

## Abstract

Long‐acting pasireotide and bromocriptine provided biochemical control of growth hormone and prolactin in a patient with plurihormonal pituitary macroadenoma, allowing near‐complete tumor excision while restoring pituitary function and avoiding adjunctive radiotherapy. Pasireotide initiation resulted in hyperglycemia, which stabilized after a few months and resolved upon pasireotide discontinuation (ACCESS; NCT01995734).

## 1 INTRODUCTION

Pituitary tumors can hypersecrete a variety of hormones, including prolactin and growth hormone (GH) leading to a global disruption of hormone balance and subsequent disease,^1^ or can be nonsecretory and diagnosed incidentally, may grow and cause symptoms due to mass effects, and should be removed if their growth is outside of the confined space of the sella.^2^ Hormonally active tumors in women are often diagnosed as microadenomas due to amenorrhea.^3^ Hormonal interference by pituitary adenomas in men is more subtle, which makes the tumors more likely to be macroadenomas at time of diagnosis.^3^ Prolactinomas are the most common hormonally active tumors and are usually amenable to therapy with dopamine agonists.^4^ These adenomas often can be “switched off” by supraphysiologic levels of dopamine and will respond with a return of serum prolactin level and gonadal function to normal and shrinkage of the tumor.^4^ However, biochemical response of tumors does not necessarily cause tumor shrinkage.^4^ In some persistent adenomas, lack of tumor shrinkage may indicate plurihormonal tumors with 1 cell line hypersecreting 2 hormones or 2 cell lines hypersecreting individual hormones.^5^

Acromegaly is a rare disorder characterized by the excess secretion of GH from a benign pituitary adenoma, which results in the overproduction of insulin‐like growth factor 1 (IGF‐1).^6^ Uncontrolled GH and IGF‐1 levels result in the development of various symptoms and comorbidities such as uncontrolled skeletal growth, soft‐tissue swelling, weight gain, headaches, excessive sweating, and sexual dysfunction. The signs of excess secretion of GH can be confused with those of other conditions such as metabolic syndrome because the bone changes can take many years to manifest.^7^ The first‐line treatment for acromegaly is resection of the underlying tumor by transsphenoidal surgery (TSS).^6^ However, TSS can be difficult to perform in cases of particularly invasive tumors, and medical therapy may need to be used.^6^, ^8^ When GH is secreted from a pituitary macroadenoma, the treatment of choice is endoscopic transsphenoidal resection of the tumor.^9^ Unfortunately, with an experienced surgeon, only 63% of patients are cured with this treatment.^9^ Somatostatin analogs (SSAs) are considered the first‐line medical treatment for acromegaly; they act by inhibiting the release of a variety of hormones, including GH.^6^, ^8^ In patients with mild disease or those who are unresponsive to SSAs, dopamine agonists and GH receptor antagonists can be used.^6^ Notably, dopamine agonists (eg, cabergoline, bromocriptine) can also inhibit the release of prolactin and may be particularly beneficial in patients with plurihormonal tumors that secrete both GH and prolactin.^6^ While some GH‐secreting adenomas have been reported to respond to cabergoline, the SSAs lanreotide and octreotide are considered cornerstone therapies to decrease tumor size.^6^ In a study by Karavitaki et al,^10^ lanreotide decreased tumor size by 20%, allowing for easier resection of GH‐secreting macroadenomas. Lanreotide and octreotide act by inhibiting the release of several hormones including GH. In patients who are unresponsive to SSAs, dopamine agonists may be added in combination.^6^

Medical therapy can prevent the growth of pituitary tumors and provide relief from symptoms due to the compressive mass effect, and it may cause tumors to shrink.^6^ Various reports have shown that the SSAs lanreotide and octreotide effectively shrink tumors and allow for easier resection by TSS.^11^ Long‐acting pasireotide is a next‐generation, multi‐receptor‐targeted SSA that is approved by the US Food and Drug Administration for the treatment of acromegaly in patients who had an inadequate response to surgery or for whom surgery is not an option.^8^ In a 12‐month Phase 3 trial (C2305; ClinicalTrials.gov identifier: NCT00600886), long‐acting pasireotide provided biochemical control in 31% of medically naive patients and decreased mean tumor volume by 40%.^12^ In the extension phase of the C2305 trial, 75% of patients treated with pasireotide for up to 25 months achieved a significant reduction in tumor volume (>20%), and the mean time to significant tumor volume reduction was 25.0 weeks.^13^ In a 24‐month Phase 3 study of patients with acromegaly that was inadequately controlled with octreotide or lanreotide (PAOLA; ClinicalTrials.gov identifier: NCT01137682), long‐acting pasireotide effectively provided biochemical control (GH <2.5 μg/L and normalization of IGF‐1 level) and/or tumor volume reduction in a subset of patients.^14^ Additionally, these studies showed that pasireotide has a similar safety profile to other SSAs, except that pasireotide is associated with elevated levels of fasting plasma glucose (FPG) and glycated hemoglobin (HbA_1c_).^12^, ^13^, ^14^

ACCESS (ClinicalTrials.gov identifier: NCT01995734) was an open‐label, uncontrolled, expanded‐treatment protocol safety study that provided patients with acromegaly access to long‐acting pasireotide before pasireotide was approved and made commercially available. Here, we present a patient with a plurihormonal pituitary macroadenoma secreting prolactin and GH, which was initially unresectable. This patient was enrolled in ACCESS, and his tumor was pharmacologically debulked with the combination of long‐acting pasireotide and bromocriptine, allowing for near‐total endoscopic resection and prolonged remission from acromegaly using only low‐dose cabergoline. Preoperative glucose intolerance and weight gain issues resolved after pharmacologic and surgical treatment, and normal pituitary function was restored.

## 2 CASE HISTORY/EXAMINATION

**Figure 1:**
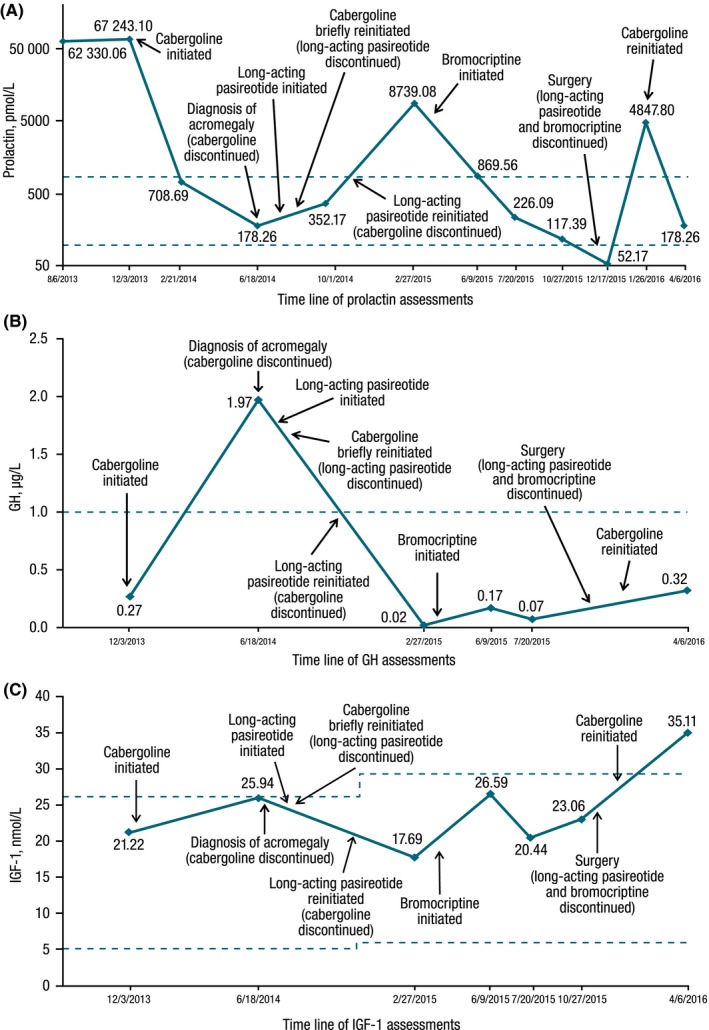
Levels of (A) prolactin, (B) GH, and (C) IGF‐1. Broken blue lines indicate the reference range for prolactin (2.1‐17.1 μg/L), the diagnostic threshold level for acromegaly for GH (>1.0 μg/L),^6^ and the age‐normalized reference range for IGF‐1 (35‐196 μg/L), respectively. GH, growth hormone; IGF‐1, insulin‐like growth factor 1

**Figure 2:**
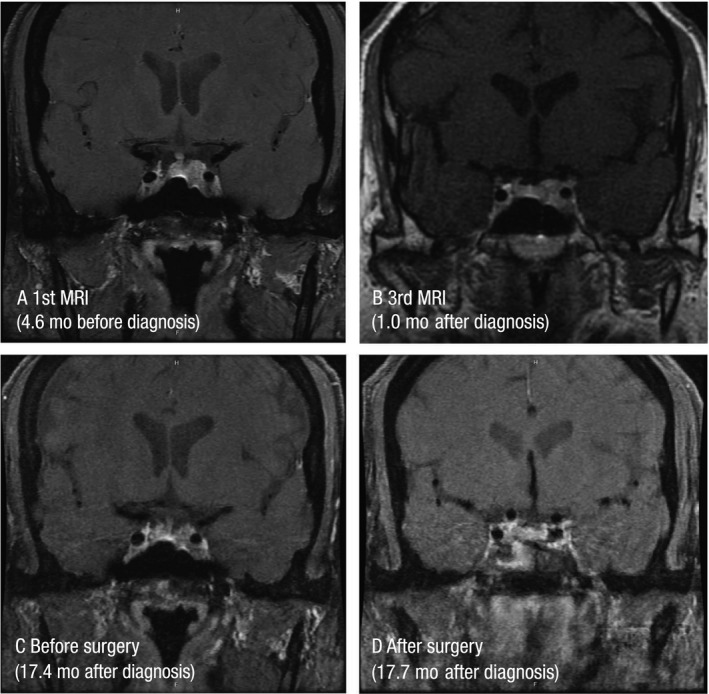
Magnetic resonance imaging of pituitary adenoma relative to the time of diagnosis and treatment of acromegaly: (A) 1st MRI, (B) 3rd MRI, (C) before surgery, and (D) after surgery. MRI, magnetic resonance imaging

A 62‐year‐old white man was referred to the endocrinology department because of low testosterone levels. He had been started on exogenous testosterone without evaluation of pituitary function and had a poor response to testosterone therapy, including no improvement in libido. He had been withdrawn from replacement therapy prior to referral. He had a history of progressive 13.6‐kg weight gain, fatigue, sleep apnea, excessive sweating, erectile dysfunction, glucose intolerance, and low‐grade headaches and also a history of severe gastroesophageal reflux disease that was subsequently found to be due to gallstone disease. He had Hashimoto thyroiditis with elevated thyroid‐stimulating hormone (TSH) and a family history hypothyroidism and type 2 diabetes. Levels of testosterone, follicle‐stimulating hormone, and luteinizing hormone were low. The prolactin level was markedly elevated (1546.6 μg/L; reference range [RR], 2.1‐17.1 μg/L; Figure 1A). Initial GH was 0.27 μg/L (RR, 0.00‐3.00) and IGF‐1 was 126 μg/L (RR, 35‐196). Magnetic resonance imaging (MRI) revealed a 3.6‐cm^3^ left‐sided pituitary adenoma with partial invasion of the left cavernous sinus and partial encapsulation of the left internal carotid artery. There was effacement of the optic chiasma (Figure 2A) with a minimal visual field cut.

Pituitary surgery is performed via a direct endoscopic, endonasal approach using modern, frameless stereotactic navigation and skull base surgery techniques. Attempting surgical resection of tumor extension lateral to the cavernous carotid artery requires an expanded approach and operating within the confines of the cavernous sinus. Contained within the cavernous sinus are the carotid artery and cranial nerves III, IV, VI, V1, and V2, which leaves them at risk for injury during surgery. To minimize the chance of postoperative cranial nerve deficit, highly specialized electromyography monitoring is required to allow for neural stimulation within the sinus to identify the location of these nerves. In order to achieve this, immediate preoperative placement of needle electrodes within the extraocular muscles is done, which puts surrounding structures at risk. Additionally, operating within the cavernous sinus greatly increases the risks of carotid injury and persistent venous blood loss potentially requiring transfusion; it also greatly increases operative and anesthesia time. Prior to biochemical debulking, this patient would have required all of these additional measures. While the risks may be warranted in select individual cases with pathologic considerations that have limited options, in most instances, it is difficult to justify these risks regarding pituitary macroadenomas without visual compromise. The currently accepted treatment paradigm is to surgically resect an easily accessible tumor and leave stereotactic radiosurgery to treat the residual disease. This option can result in radiation‐induced cranial nerve injury and will result in hypopituitarism.

## 3 INVESTIGATIONS AND TREATMENT

The patient was given cabergoline, with the expectation that prolactin levels would be suppressed, normal testosterone function would return, and the tumor would shrink. The initial dose was 250 μg twice weekly (Table 1). After 2 months of therapy, the prolactin levels had normalized (16.3 μg/L; RR, 2.1‐17.1 μg/L), testosterone function had returned to normal, and weight had decreased from 128.4 to 120.2 kg, but tumor volume (3.5 cm^3^) was not affected. The cabergoline dose was titrated to 500 μg twice weekly, and the patient developed orthostatic hypotension. Consequentially, cabergoline dosage was reduced to 250 μg twice weekly, and gradually with improved tolerance of this dose, cabergoline therapy was changed to 250 μg every other day.

**Table 1:** Summary of treatment

Visit	Time since diagnosis of acromegaly, mo	Medication	Tumor volume, cm^3^
First MRI (Figure 2A)	−4.6	Cabergoline 250 μg twice weekly	3.6
Second MRI	−1.8	Cabergoline 500 μg twice weekly (then changed to 250 μg twice weekly and to 250 μg every other day)	3.5
Acromegaly diagnosis	0.0	Long‐acting pasireotide 40 mg IM monthly	ND
Third MRI (Figure 2B)	1.0	Long‐acting pasireotide 40 mg IM monthly, bromocriptine 2.5 mg at bedtime (bromocriptine then increased to 5 mg at bedtime)	2.7
Before surgery (Figure 2C)	17.4	No changes	1.7
After surgery (Figure 2D)	17.7	None	0

IM, intramuscular; MRI, magnetic resonance imaging; ND, not determined.

After 6 months of cabergoline treatment, prolactin levels remained in the low RR (4.1 μg/L; RR, 3.1‐17.1 μg/L) and weight had further decreased to 117.5 kg. There was no improvement in glucose tolerance, hypertension, excess sweating, and headaches. Evaluation for acromegaly revealed hypersecretion of GH (2.0 μg/L; Figure 1B) and IGF‐1 (198 μg/L; RR, 35‐196 μg/L;Figure 1C). The ACCESS trial was available in our center and required only IGF‐1 elevation and not failure of GH to suppress on oral glucose tolerance test; therefore, such a test was not performed. This decision did not affect his clinical course since he was already committed to surgery for an invasive macroadenoma.

Upon enrollment in the ACCESS study, the patient underwent an 8‐week cabergoline washout period followed by treatment with long‐acting pasireotide 40 mg, per study protocol. Within the first month of treatment with pasireotide, the patient reinitiated cabergoline and temporarily discontinued pasireotide in response to increasing headaches. Following another 8‐week cabergoline washout period, pasireotide was reinitiated.

Three months after reinitiating pasireotide, the patient's GH level had reduced to 0.02 μg/L and IGF‐1 level had normalized (135 μg/L; RR, 46‐219 μg/L). Although the tumor volume had decreased to 2.7 cm^3^, the tumor remained invasive. Additionally, the prolactin level had increased 201 μg/L (RR, 2.1‐17.1 μg/L). The ACCESS study sponsor granted permission to deviate from the protocol by initiating bromocriptine 2.5 mg once daily in an attempt to decrease prolactin levels. Bromocriptine did not prolong the QT interval. After 7 months of combination therapy with bromocriptine and pasireotide, the patient was transitioned off the ACCESS study to commercial pasireotide 40 mg.

Eight months after initiating combination therapy, the patient's IGF‐1 level remained normalized at 176 μg/L (RR, 46‐219 μg/L), prolactin levels were normalized, and tumor volume was reduced to 1.7 cm^3^. A preoperative evaluation indicated the patient had a fasting cortisol level of 215.2 nmol/L. Twenty‐four‐hour urinary free cortisol was 5.54 μg/g cortisone. This was lower than his pretreatment cortisol level, which possibly was a side effect of pasireotide therapy, but this has not been previously described. The patient was placed on prednisone 5 mg, taken every morning, and preoperative steroid coverage was ordered. Subsequently, TSS was performed to resect the tumor, which was easily dissected from surrounding structures. It is likely this outcome was greatly enhanced by preoperative pharmacologic debulking of the tumor, allowing the inherent risks associated with operating within the deeper portions of the cavernous sinus to be avoided. There was a surgical operating time of 208 minutes from initiation of lumbar drain placement to the removal of the MAYFIELD^®^ skull clamp. Blood loss was estimated at 200 mL, and the patient had an uncomplicated postoperative course. Immunohistochemical staining was strongly and diffusely positive for prolactin, whereas only isolated cells were positive for GH. No staining was seen with antibodies to adrenocorticotropic hormone, follicle‐stimulating hormone, and luteinizing hormone.

## 4 OUTCOME AND FOLLOW‐UP

After surgery, no residual tumor was visible by MRI, (Figure 2D) and the patient's weight decreased to 113.0 kg. He had transient diabetes insipidus, which resolved prior to discharge. Long‐acting pasireotide and bromocriptine treatment were discontinued. Two months after surgery, the patient's prolactin level had increased to 111.5 μg/L (RR, 2.1‐17.1 μg/L), and cabergoline was reinitiated. After 2 months of cabergoline treatment, the prolactin level had decreased to 4.1 μg/L (RR, 2.1‐17.1 μg/L). However, the IGF‐1 level had increased to 268 μg/L (RR, 46‐219 μg/L) and the GH level had increased to 0.32 μg/L. The patient had a follow‐up MRI that showed some residual tumor in the wall of the cavernous sinus. An oral glucose tolerance test was performed, which revealed normal glucose tolerance and complete suppression of GH. Therefore, cabergoline was increased to 250 μg twice weekly. There were no signs of adrenal insufficiency, and slow taper of prednisone was started. The patient was weaned off all hormone therapy except for levothyroxine and cabergoline. At 18 months after surgery, the prolactin level was 0.34 μg/L, GH was 0.08 μg/L, HbA_1c_ was 5.7%, and testosterone level was 9.76 nmol/L (RR, 7.1‐27.1 nmol/L). Steroid replacement was discontinued, with a fasting cortisol of 253.8 nmol/L; TSH was 1.65 mU/L with free T4 of 0.94 ng/dL. The patient was asymptomatic and had returned to full‐time work.

At the time of enrollment in the study, HbA_1c_, FPG level, and insulin level was 5.9%, 5.9 mmol/L, and 20 mIU/mL, respectively (Table 2). When long‐acting pasireotide was reinitiated after the brief discontinuation, HbA_1c_, FPG level, and insulin level was 6.2%, 6.3 mmol/L, and 24 mIU/mL, respectively. One month after therapy with pasireotide was reinitiated, the FPG level had increased to 7.1 mmol/L, and the insulin level had decreased to 13 mIU/mL. The FPG level peaked at 7.8 mmol/L 3 months after reinitiation of pasireotide, when the insulin level was 10 mIU/mL. Two months later, the FPG level had decreased to 6.6 mmol/L, and the insulin level had increased to 12 mIU/mL. During treatment with pasireotide, HbA_1c _peaked at 6.4%. Eighteen months after treatment, HbA_1c_ had decreased to 5.7% in this patient with a family history of type 2 diabetes mellitus. No antidiabetic medications were taken during the course of observation.

**Table 2:** Glycemic levels

Time since diagnosis of acromegaly, mo	HbA_1c_, %	FPG, mmol/L	Insulin, pmol/L
0.2	5.9	5.9	138.9
5.1	6.2	6.3	166.7
6.3		7.1	90.3
7.3		7.1	83.3
8.5		7.8	69.5
10.5		6.6	83.3
11.9	6.4	6.6	90.3
25.9	5.9		

FPG, fasting plasma glucose; HbA_1c_, glycated hemoglobin.

## 5 DISCUSSION

Here, we present a complicated case of a pituitary macroadenoma secreting prolactin and GH from 2 cell lines. Approximately 25% of GH‐secreting adenomas co‐secrete prolactin,^15^ and co‐secretion of GH in prolactinomas has been documented at the time of initial diagnosis and years afterward.^16^ MRI (Figure 2D) and observation during this patient's surgery did not reveal separate tumors, and the bulk of the tumor resected was secreting prolactin with sporadic cells secreting GH. This patient had no family history of multiple endocrine neoplasia (MEN) and no other hormonal abnormalities suggesting MEN. Co‐secreting adenomas include dimorphic adenomas composed of both GH‐ and prolactin‐secreting cells, monomorphous mammosomatotroph adenomas, and, rarely, stem‐cell acidophilic adenomas. In this patient, single‐agent treatment with the potent dopamine agonist cabergoline reduced prolactin levels to the normal range but did not cause substantial tumor shrinkage. Single‐agent treatment with pasireotide suppressed GH and IGF‐1 levels and had some effect on prolactin level in that it increased to only 201 µg/L rather than >1500 µg/L prior to cabergoline treatment. The tumor size decreased with single‐agent pasireotide therapy, but the tumor remained invasive. Long‐acting pasireotide and bromocriptine combination therapy provided dual biochemical control with a substantial decrease in tumor volume. Endoscopic resection was possible with minimal residual tumor in the left cavernous sinus. Preoperative pituitary function was impaired before surgery and completely returned to normal after surgery. There was no evidence of tumor regrowth on sequential blood sampling and MRI scanning (Figure 2D). This patient's outcome was greatly enhanced by preoperative pharmacologic debulking of the tumor, thereby avoiding the inherent risks associated with operating within the deeper portions of the cavernous sinus.

Long‐acting pasireotide is a next‐generation SSA indicated for the treatment of acromegaly in patients who did not respond adequately to surgery or for whom surgery is not an option.^8^ In clinical trials, pasireotide decreased tumor volume in most patients at rates similar to those observed for other SSAs.^12^, ^13^ These findings suggest that long‐acting pasireotide may also be useful for presurgical debulking of GH‐secreting tumors. Dopamine agonists are indicated for the presurgical debulking of macroadenomas secreting prolactin,^17^ but there are no treatment protocols for the presurgical debulking of tumors secreting both prolactin and GH that fail to shrink in response to dopamine agonists. Preclinical data demonstrated that long‐acting pasireotide inhibits the hypersecretion of both prolactin and GH from isolated prolactin‐ and GH‐secreting tumor cells more strongly than octreotide.^18^ Pasireotide is a more potent activator of somatostatin receptor 5 (sst_5_) than octreotide, and sst_5_ is the predominantly expressed sst in most prolactin‐secreting tumors, suggesting that long‐acting pasireotide may be useful in the treatment of prolactin‐ and GH‐secreting tumors.^18^, ^19^ This case also demonstrates that failure of prolactinomas to shrink in response to potent SSAs should indicate reevaluation for co‐secretion of GH. If this combination had not allowed successful presurgical debulking, it is likely the patient would have been referred for radiation therapy, which would have caused permanent hypopituitarism and put the patient at risk for cranial nerve injury.

In clinical trials, FPG levels and HbA_1c_ increased after long‐acting pasireotide therapy was initiated and peaked after several months before stabilizing.^12^, ^13^, ^14^ Consistent with these results, the patient in this case study had an initial decrease in insulin levels, which then stabilized after several months. Additionally, his HbA_1c_ increased after initiation of long‐acting pasireotide and dropped to baseline levels after discontinuation. No serious adverse events were observed. Taken together, these data exemplify the safe and effective use of long‐acting pasireotide and bromocriptine combination therapy in the treatment of acromegaly with concomitant prolactin hypersecretion. This case study on preoperative debulking of pituitary tumors is an important addition to the literature as an alternative strategy for hard‐to‐reach skull base locations.

## CONFLICT OF INTEREST

TJ is on the Speakers Bureau for Novo Nordisk, AstraZeneca, Jansen and AbbVie. RJ is on the Speakers Bureau for Novo Nordisk. RF has no conflict of interest to declare.

## AUTHOR CONTRIBUTION

TJ: was the physician who diagnosed the patient and began treatment. RJ: was the study coordinator for the clinical trial and was involved in modifying other drug therapies to avoid interactions. RF: was the neurosurgeon who operated on him. All co‐authors contributed to the concept, draft development, and approval of the final manuscript for submission.
